# Caution in using second generation tyrosine kinase inhibitor, especially for first line therapy of chronic myeloid leukemia

**DOI:** 10.1002/ajh.26618

**Published:** 2022-06-09

**Authors:** Carlo Gambacorti‐Passerini, Franck Emmanuel Nicolini, Richard A. Larson, Andrea Aroldi, Diletta Fontana, Rocco Piazza, Philipp le Coutre, Laura Antolini, Sarit Assouline

**Affiliations:** ^1^ Hematology Division ASST Monza Monza Italy; ^2^ University of Milano‐Bicocca Monza Italy; ^3^ Hematology Department & INSERM U1052 Centre Léon Bérard Lyon France; ^4^ University of Chicago Chicago Illinois USA; ^5^ Department of Hematology, Oncology, and Tumor Immunology Charité ‐ Universitätsmedizin Berlin Berlin Germany; ^6^ Jewish General Hospital McGill University Montreal Quebec Canada

To the Editor:

A normal life expectancy for chronic myeloid leukemia (CML) patients treated with tyrosine kinase inhibitors (TKIs) was first identified in 2011 and 2014 as a result of clinical trials,^1^, ^2^, ^3^ and later confirmed by Bower et al. at the level of a nationwide tumor registry.^4^ The same Swedish group now presents data on morbidities in the same CML patient population.^5^ Their results are important and deserve attention. However, part of the data require further discussion and some raise doubts. Rates of 142 nominal disease categories were significantly increased in CML patients versus the general population, even after excluding the initial 6 months of treatment, when factors associated with the presence of uncontrolled leukemia could be involved in generating the reported abnormalities. Second cancers are notably absent since the authors decided to exclude them, while an excess of cardiovascular diseases (CVD) is present. This fact is surprising for several reasons:

Patients with a cancer diagnosis in general^6^ and those with Chronic Myeloproliferative diseases (CMPD) in particular, are known to have an increased incidence of second neoplasias when compared to the general population. Landtblom and colleagues^7^ using the same Swedish registry found an increase of 60% in the incidence of second cancers, particularly skin, kidney, brain, pancreas, lung, head and neck, endocrine cancers, and malignant melanoma in Ph‐negative CMPD, Frederiksen et al,^8^ using a Danish registry, identified an increased risk of second cancers in CMPD patients including CML patients, in whom the relative risk was 1.6. Rebora and colleagues^9^ using the Swedish database but in the pre‐imatinib era reported an increased risk of stomach, skin, urogenital tract cancers, and lymphoid leukemia for CML patients. Based on these data our general policy for CML patients is to actively look for early diagnosis of second cancers. Whether this result derives from increased susceptibility, increased monitoring of patients or both, it remains to be established.

Imatinib, the most frequently used TKI, has not been associated with increased risk of CVD, rather it was even suggested to be “cardioprotective.”^10^ This could be linked to the preferential inhibition of PDGFR over ABL1 operated by imatinib.

Could it be that the apparent absence of neoplastic diseases in the present analysis from Bower originates from a relative but artificial increase of CVD incidence due to a possible direct dependence between time to second neoplasia and CVD?

A second important aspect of this paper relates to the use of second generation TKI (2GEN). The report from Dahlén identified several serious and potentially fatal adverse events that were significantly increased in patients treated with 2GEN when compared to imatinib users, utilized here as a benchmark. This was particularly evident for nilotinib and dasatinib, for which sufficient data were available, while insufficient data were present regarding the use of bosutinib and ponatinib.

Not surprisingly, Nilotinib resulted in an increased risk of cardiovascular events (myocardial infarction, hypertension, atherosclerosis, chronic myocardial disease) and diabetes development, while dasatinib use showed increased risks of pleural effusions and infections. Since CML patients may be taking these TKIs for many years, it is incumbent on physicians to manage and minimize treatment‐related risks and co‐morbid conditions.

Given these results and the fact that 2GEN failed to substantially decrease the risk of CML progression to accelerated phase/blast crisis, when tested against imatinib in more than 15 controlled studies in the first line setting (Table 1),^11^ extreme caution should be exercised when deciding to use 2GEN, especially for the first line treatment of chronic phase CML patients. It is reassuring to see that imatinib remains the most frequently prescribed TKI for CML over the time analyzed in this study. Risk assessment using the Sokal or ELTS score can identify the low‐ and intermediate‐risk patients who definitely do not require initial treatment with a 2GEN. Imatinib constitutes an efficient, safe, and considerably less expensive first line CML treatment option. Its cost will undoubtedly constitute an additional advantage in countries with limited resources to assure treatment availability without financial restrictions. 2GEN definitely fulfill an important role in the treatment armamentarium, but are best used in second or subsequent lines of treatment, and according to their ability to cover drug‐resistant mutations. Several physicians also use 2GEN initially for high‐risk patients; while this use is frequent, no controlled study or a sub‐analysis of it ever documented a statistically significant difference in this subgroup when compared to imatinib. When dealing with high‐risk patients a close monitoring of the patient clinical course is probably the best strategy, in order to shift TKI or to proceed to BMT without delay. Interestingly, the use of nilotinib peaked from 2011 to 2013 in the Dahlén study, when this drug was aggressively marketed for first line treatment of CML, but then dropped in subsequent years, perhaps reflecting the emerging data of increasing CVD risk with time. It is also true that 2GEN generally lead to faster decrease in minimal residual disease; however, curves come close with time, and the failure rate of treatment discontinuation after imatinib or 2GEN is not different.^12^

**TABLE 1:** A summary of controlled studies performed to evaluate the first line treatment of CML.

Trial	Drugs	# of patients enrolled	References (listed here below)
IRIS	IM 400 vs IFN/AraC	1106	(a)
TOPS	IM 400 vs IM 800	476	(b)
GIMEMA	IM 400 vs IM 800	216	(c)
SWOG	IM 400 vs IM 800	153	(d)
DASISION	IM 400 vs DAS 100	519	(e)
SWOG 0325	IM 400 vs DAS 100	253	(f)
SPIRIT	IM 400 ± AraC or ± PegIFN vs IM 600	636	(g)
CML IV	IM 400 ± IFN vs IM/400 + AraC vs IM 800	1536	(h); (i)
ENESTnd	IM 400 vs NIL 600 vs NIL 800	846	(l)
ENEST China	NIL 300 BID vs Imatinib 400	267	(m)
BFORE	IM 400 vs BOS 400	536	(n)
BELA	IM 400 vs BOS 500	502	(o)
SPIRIT 2	IM 400 vs DAS 100	812	(p)
EPIC	IM 400 vs Ponatinib 45	307	(q)
Radotinib	IM 400 vs. Radotinib 300 BID	241	(r)

*Note*: Studies with drug name marked in red are the ones which led to drug registration for frontline use. [a] N Engl J Med. 2017;376(10):917–927. doi: 10.1056/NEJMoa1609324. [b] J Clin Oncol. 2010;28(3):424–430. doi: 10.1200/JCO.2009.25.3724. [c] Blood. 2009;113(19):4497–4504. doi: 10.1182/blood‐2008‐12‐191254. [d] Br J Haematol. 2014;164(2):223–232. doi: 10.1111/bjh.12618. [e] N Engl J Med. 2010;362(24):2260–2270. doi: 10.1056/NEJMoa1002315. [f] Blood. 2012;120(19):3898–3905. doi: 10.1182/blood‐2012‐02‐410688. [g] N Engl J Med. 2010;363(26):2511–2521. doi: 10.1056/NEJMoa1004095. [h] Leukemia. 2015;29(5):1123–1132. doi: 10.1038/leu.2015.36. [i] Leukemia. 2017;31(11):2398–2406. doi: 10.1038/leu.2017.253. [l] N Engl J Med. 2010;362(24):2251–2259. doi: 10.1056/NEJMoa0912614. [m] Blood. 2015;125(18):2771–2778. doi: 10.1182/blood‐2014‐09‐601674. [n] J Clin Oncol. 2018;36(3):231–237. doi: 10.1200/JCO.2017.74.7162. [o] J Clin Oncol. 2012;30(28):3486–3492. doi: 10.1200/JCO.2011.38.7522. [p] Haematologica. 2015;100:182. [q] Lancet Oncol. 2016;17(5):612–621. doi: 10.1016/S1470‐2045(16)00080‐2. [r] Blood 2015;126(23):476. doi: 10.1182/blood.V126.23.476.476.

In conclusion, the report from Bower et al. highlights an important issue in the management of CML: when a normal life expectancy is the goal of the therapy, the safety of the chosen TKI becomes of paramount importance, especially for first line therapy. Further research on the incidence of second cancers and of CVD in the entire cohort of CML patients is still needed.

## FUNDING INFORMATION

Funded in part from AIRC under IG 2017 ‐ ID. 20112 project—P.I. Gambacorti‐Passerini Carlo.

## CONFLICT OF INTEREST

Richard A. Larson has acted as a consultant or advisor to Amgen, Ariad/Takeda, Astellas, Celgene/Bristol Myers Squibb, CVS/Caremark, Epizyme, Immunogen, MedPace, MorphoSys, Novartis, and Servier, and has received clinical research support to his institution from Astellas, Celgene, Cellectis, Daiichi Sankyo, Forty Seven/Gilead, Novartis, Rafael Pharmaceuticals, and royalties from UpToDate. The other authors declare no potential conflict of interests.

## Data Availability

Data sharing is not applicable to this article as no new data were created or analyzed in this study.

## References

[1] Gambacorti‐Passerini C , Antolini L , Mahon FX , et al. Multicenter independent assessment of outcomes in chronic myeloid leukemia patients treated with imatinib. J Natl Cancer Inst. 2011;103(7):553‐561. doi:10.1093/jnci/djr060 21422402

[2] Hehlmann R , Muller MC , Lauseker M , et al. Deep molecular response is reached by the majority of patients treated with imatinib, predicts survival, and is achieved more quickly by optimized high‐dose imatinib: results from the randomized CML‐study IV. J Clin Oncol. 2014;32(5):415‐423. doi:10.1200/JCO.2013.49.9020 24297946

[3] Vigano I , Di Giacomo N , Bozzani S , et al. First‐line treatment of 102 chronic myeloid leukemia patients with imatinib: a long‐term single institution analysis. Am J Hematol. 2014;89(10):E184‐E187. doi:10.1002/ajh.23804 25041880

[4] Bower H , Bjorkholm M , Dickman PW , et al. Life expectancy of patients with chronic myeloid leukemia approaches the life expectancy of the general population. J Clin Oncol. 2016;34(24):2851‐2857. doi:10.1200/JCO.2015.66.2866 27325849

[5] Dahlén T , Edgren G , Ljungman P , et al. Adverse outcomes in chronic myeloid leukemia patients treated with tyrosine kinase inhibitors: follow‐up of patients diagnosed 2002‐2017 in a complete coverage and nationwide agnostic register study. Am J Hematol. 2022;97:421‐430. doi:10.1002/ajh.26463 35015312 PMC9306877

[6] Curtis RE , Freedman DM , Ron E , et al. New Malignancies Among Cancer Survivors: SEER Cancer Registries, 1973–2000. National Cancer Institute. NIH Publ. No. 05–5302; 2006.

[7] Landtblom AR , Bower H , Andersson TM‐L , et al. Second malignancies in patients with myeloproliferative neoplasms: a population‐based cohort study of 9379 patients. Leukemia. 2018;32(10):2203‐2210. doi:10.1038/s41375-018-0027-y 29535425 PMC7552081

[8] Frederiksen H , Farkas DK , Christiansen CF , et al. Chronic myeloproliferative neoplasms and subsequent cancer risk: a Danish population‐based cohort study. Blood. 2011;118(25):6515‐6520. doi:10.1182/blood-2011-04-348755 22039256

[9] Rebora P , Czene K , Antolini L , et al. Are chronic myeloid leukemia patients more at risk for second malignancies? A population‐based study. Am J Epidemiology. 2010;172(9):1028‐1033. doi:10.1093/aje/kwq262 20861143

[10] Aghel N , Delgado DH , Lipton JH . Cardiovascular toxicities of BCR‐ABL tyrosine kinase inhibitors in chronic myeloid leukemia: preventive strategies and cardiovascular surveillance. Vasc Health Risk Manag. 2017;13:293‐303. doi:10.2147/VHRM.S108874 28831263 PMC5552150

[11] Gambacorti‐Passerini C , le Coutre P . Chronic Myeloid Leukemia. DeVita, Hellman, and Rosenberg's Cancer: Principles & Practice of Oncology. Lippincott Williams & Wilkins Publisher; 2018:1773‐1784.

[12] Inzoli E , Aroldi A , Piazza R , Gambacorti‐Passerini C . Tyrosine kinase inhibitor discontinuation in chronic myeloid leukemia: eligibility criteria and predictors of success. Am J Hematol. Published online April 05, 2022. doi:10.1002/ajh.26556 PMC9546318 35384030

